# Immunization by Replication-Competent Controlled Herpesvirus Vectors

**DOI:** 10.1128/JVI.00616-18

**Published:** 2018-07-31

**Authors:** David C. Bloom, Robert K. Tran, Joyce Feller, Richard Voellmy

**Affiliations:** a Department of Molecular Genetics and Microbiology, University of Florida College of Medicine, Gainesville, Florida, USA; b HSF Pharmaceuticals SA, La Tour-de-Peilz, Switzerland; c Department of Physiological Sciences, University of Florida College of Veterinary Sciences, Gainesville, Florida, USA; Northwestern University

**Keywords:** herpesvirus vector, immunization, vaccination, regulation of viral genes, regulation of viral replication, two-component gene switch

## Abstract

Replication-competent controlled virus vectors were derived from the virulent herpes simplex virus 1 (HSV-1) wild-type strain 17*syn+* by placing one or two replication-essential genes under the stringent control of a gene switch that is coactivated by heat and an antiprogestin. Upon activation of the gene switch, the vectors replicate in infected cells with an efficacy that approaches that of the wild-type virus from which they were derived. Essentially no replication occurs in the absence of activation. When administered to mice, localized application of a transient heat treatment in the presence of systemic antiprogestin results in efficient but limited virus replication at the site of administration. The immunogenicity of these viral vectors was tested in a mouse footpad lethal challenge model. Unactivated viral vectors—which may be regarded as equivalents of inactivated vaccines—induced detectable protection against lethality caused by wild-type virus challenge. Single activation of the viral vectors at the site of administration (rear footpads) greatly enhanced protective immune responses, and a second immunization resulted in complete protection. Once activated, vectors also induced far better neutralizing antibody and HSV-1-specific cellular immune responses than unactivated vectors. To find out whether the immunogenicity of a heterologous antigen was also enhanced in the context of efficient transient vector replication, a virus vector constitutively expressing an equine influenza virus hemagglutinin was constructed. Immunization of mice with this recombinant induced detectable antibody-mediated neutralization of equine influenza virus, as well as a hemagglutinin-specific cellular immune response. Single activation of viral replication resulted in a severalfold enhancement of these immune responses.

**IMPORTANCE** We hypothesized that vigorous replication of a pathogen may be critical for eliciting the most potent and balanced immune response against it. Hence, attenuation/inactivation (as in conventional vaccines) should be avoided. Instead, the necessary safety should be provided by placing replication of the pathogen under stringent control and by activating time-limited replication of the pathogen strictly in an administration region in which pathology cannot develop. Immunization will then occur in the context of highly efficient pathogen replication and uncompromised safety. We found that localized activation in mice of efficient but limited replication of a replication-competent controlled herpesvirus vector resulted in a greatly enhanced immune response to the virus or an expressed heterologous antigen. This finding supports the above-mentioned hypothesis and suggests that the vectors may be promising novel agents worth exploring for the prevention/mitigation of infectious diseases for which efficient vaccination is lacking, in particular in immunocompromised patients.

## Abstract (precis)

We hypothesized that vigorous replication of a pathogen may be critical for eliciting the most potent and balanced immune response against it. Hence, attenuation/inactivation (as in conventional vaccines) should be avoided. Instead, the necessary safety should be provided by placing replication of the pathogen under stringent control and by activating time-limited replication of the pathogen strictly in an administration region in which pathology cannot develop. Immunization will then occur in the context of highly efficient pathogen replication and uncompromised safety. We found that localized activation in mice of efficient but limited replication of a replication-competent controlled herpesvirus vector resulted in a greatly enhanced immune response to the virus or an expressed heterologous antigen. This finding supports the above-mentioned hypothesis and suggests that the vectors may be promising novel agents worth exploring for the prevention/mitigation of infectious diseases for which efficient vaccination is lacking, in particular in immunocompromised patients.

## INTRODUCTION

Vaccination has been spectacularly successful, but there remain important infections for which therapeutic or preventative vaccines either do not exist or are of insufficient efficacy. One such example is infections caused by herpes simplex virus 1 (HSV-1) and HSV-2. Recent estimates place the worldwide prevalence of HSV-1 at 67% and that of HSV-2 at 11% (1, 2). Currently, there is no vaccine for treating or preventing HSV-1 or HSV-2 infections. Several new candidate vaccines have been developed in recent years, some of which have entered clinical evaluation (3–7). Another example is influenza virus infections. Annual infection rates for influenza virus are between 5 and 20%, and the yearly death rate worldwide lies between 250,000 and 500,000 according to WHO estimates. At this time, the vaccines remain seasonal, i.e., they need to be updated/reformulated for every influenza season. Typical subunit vaccines are at least trivalent, comprising hemagglutinins (HAs) from H1N1 and H3N2 influenza A virus strains and an influenza B virus strain (referred to as TIV). More recently, live attenuated influenza virus vaccines (LAIV) were introduced. An updated systematic review and meta-analysis published in 2012 documented a mean efficacy for TIV of 62% against real-time (RT) PCR- or virus-confirmed influenza (all age groups) (8). Limited data were available for adults 65 years of age and older. The mean efficacy for LAIV in young children was higher, but little or no protection was found in adults (18 years and older). A recent study reported a mean overall vaccine efficacy of 48% for the 2015-2016 season and lower efficacies for persons 50 years of age and older (9). Thus, currently available influenza vaccines provide moderate protection against virologically confirmed disease, but the protection is not long-lasting (10) and is especially tenuous in the senior population. Ongoing research is aimed at developing vaccines that elicit more potent antibody or T cell responses to conserved viral epitopes (11–13). A third example concerns Mycobacterium tuberculosis infections, which affect about one-third of the world population, with yearly death rates of over 1 million. Of concern in the industrialized world is the rise of drug-resistant forms of the disease (14). The only vaccine in use is the (live) bacillus Calmette-Guérin (BCG) vaccine, whose efficacy appears to be variable in adults. Candidate vaccines currently under development include virus-vectored vaccines presenting BCG antigens, adjuvanted subunit vaccines, and whole-cell vaccines (15–17). Further examples include malaria and HIV/AIDS. As these examples reveal, adequate protection against many important infections is not yet available. Research continues, and new immunization approaches appear to be urgently needed.

The question of whether live attenuated vaccines that have retained some capacity for replication provide more robust protective immunity than inactivated nonreplicating vaccines was already debated more than 50 years ago (18–21). Several more recent studies attempted to address the issue directly. One such study compared immune responses to an HIV envelope antigen expressed from an attenuated replicating vector and a replication-defective adenovirus vector in a chimpanzee model (22). Inoculation with the replicating recombinant resulted in a greater frequency of HIV envelope-specific interferon gamma-secreting peripheral blood lymphocytes, better priming of T cell proliferative responses, higher anti-envelope binding and neutralizing antibody titers, and better antibody-dependent cellular cytotoxicity. In another study, an attenuated vaccinia virus (MVTT) expressing the spike protein (S) of severe acute respiratory syndrome coronavirus (SARS-CoV) (MVTT-S) was compared with a replication-defective vaccinia virus (MVA) expressing the same protein (MVA-S). Intranasal or intraoral vaccination of mice with MVTT-S produced 20- to 100-fold higher neutralizing antibody levels than MVA-S (23, 24). Yet another study employed a mouse ocular challenge model to demonstrate that an attenuated replicating HSV-1 strain (ICP0^−^), but not a replication-defective HSV-1 strain (ICP4^−^), elicited a protective immune response (25). These findings support the theory that attenuated replicating viruses induce more complete and more potent immune responses to autologous or heterologous antigens than the corresponding nonreplicating viruses.

We hypothesized that a virus vector that could replicate in a controlled fashion with nearly the same efficiency as the respective wild-type virus (referred to here as a replication-competent controlled virus vector) would induce an even more potent and complete immune response to itself or an expressed heterologous antigen than a corresponding attenuated vector (26). Our hypothesis is in part based on the rational expectation that an efficiently replicating virus will produce a stronger inflammatory response than an attenuated virus and that the inflammatory response will result in potent activation of the innate immune system and, consequently, in strong and lasting B and T cell responses (27). To realize such an immunization strategy, a regulation system must be employed that reliably and stringently controls viral replication and is capable of being turned on and off at will. However, virus disseminates after administration. Simply restricting the number of replication cycles will not be enough: depending on the number of cycles allowed, there will be a more or less pronounced manifestation of the toxicity typical for the viral vector used. Hence, the regulation system must be capable of exerting regional control over viral replication so that the immunizing virus replicates only in a locale in which it is certain not to cause a disease phenotype.

**FIG 1:**
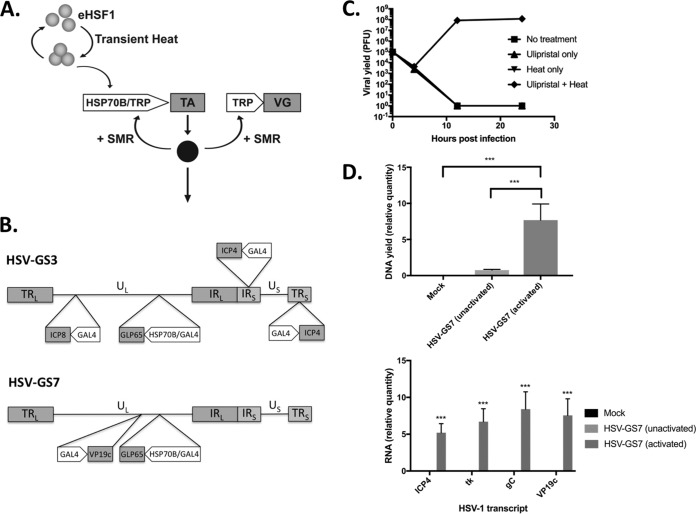
Two-component HSP70B promoter-based gene switch and replication-competent, controlled HSV-1-derived viral vectors. (A) Illustration of the operation of the dual-responsive gene switch employed to control replication of the viral vectors in the present study. The gene switch comprises a gene for a small-molecule regulator (SMR)-activated transactivator (TA). The TA gene is controlled by a promoter cassette containing an HSP70B promoter and a TA-responsive promoter (TRP). A replication-essential viral gene (VG) is under the control of a TRP. Heat treatment prompts transient activation of an endogenous heat shock transcription factor (eHSF1) that then transactivates the TA gene. TA (solid circle) is synthesized but remains inactive until activated by its SMR. Activated TA mediates expression of the viral gene and autoactivates its own expression. (B) Diagrams of viral vectors HSV-GS3 and HSV-GS7. Both vectors contain, inserted into the intragenic region between the HSV-1 UL43 and UL44 genes, a GLP65 transactivator gene functionally linked to a promoter cassette consisting of a human HSP70B promoter and a GAL4-responsive promoter. In HSV-GS3, the promoters of the replication-essential genes encoding ICP4 and ICP8 were replaced with GAL4-responsive promoters. In HSV-GS7, the promoter of the replication-essential gene for VP19c was replaced with a GAL4-responsive promoter. TR_L_ and TR_S_, long and short terminal repeats; U_L_ and U_S_, long and short unique regions; IRL and IRS, long and short internal repeats. (Panels A and B are adapted from reference 35.) (C) Single-step growth experiment for HSV-GS7 in Vero cells. Heat, exposure to 43.5°C for 30 min immediately after infection (i.e., immediately after removal of the viral inoculum); Ulipristal, 10 nM ulipristal was added to the medium at the time of initial infection. Each data point represents the mean of the results of three individual assays. Error bars are not visible due to their range. The values are PFU per milliliter. For details, see Materials and Methods. (D) Replication of HSV-GS7 (top) and expression of viral genes (bottom) in the rear feet of mice (group size [*n*] = 5) determined 24 h after administration of the recombinant vector (50,000 PFU) to the plantar surfaces of the rear feet. HSV-GS7 replication either was not activated or was activated 3 h after infection by immersion of the infected rear feet in a 45°C water bath for 10 min in the presence of ulipristal. Ulipristal (50 μg/kg) was administered i.p. at the time of initial infection. Twenty-four hours after infection, the animals were euthanized, and DNA and RNA were isolated from the infected rear feet. Levels of viral DNA were assessed by qPCR and levels of ICP4, TK, gC, and VP19c transcripts by RT-qPCR. Normalization of DNA and RNA quantities was relative to the APRT cellular gene. Relative values and standard deviations are shown. ***, *P* ≤ 0.05 for the comparison of HSV-GS7 (activated) and HSV-GS7 (unactivated) or Mock.

To facilitate construction of a replication-competent controlled virus vector, advantage may be taken of gene switches developed previously for eukaryotic cells (28). This choice implies that the backbone virus must be selected from the group of viruses having a double-stranded DNA genome (e.g., herpesviruses) or producing a double-stranded DNA intermediate during replication and utilizing the host machinery for transcription. It appears that the above-described requirements are best met by promoters of certain heat shock protein (HSP) genes that are stringently controlled by the reversibly heat-activated endogenous transcription factor HSF1 (28–30). A virus in which one or, preferably, two replication-essential genes are placed under the control of such a promoter could be activated to undergo one round of localized replication by administration of a mild heat dose to the inoculation region. Further rounds may be induced by repeated heat treatments. However, virus replication may be inefficient, because heat activation of HSP promoters is rapidly reversible, and may be unsafe, because unintended weak activation could be triggered by high fever, intoxication, etc., in an inoculated subject. We previously described a two-component HSP promoter-based gene switch that lacks these unwanted properties (31) (Fig. 1A). The first component comprises a gene for a small-molecule regulator (SMR)-activated transactivator (TA). The TA gene is controlled by a promoter cassette containing an HSP70B (HSPA7) promoter and a TA-responsive promoter (TRP). The second component is a TRP that drives a selected viral gene (VG). Heat treatment prompts expression of the TA, which is inactive unless activated by its SMR. Activated TA mediates expression of the viral gene and autoactivates its own expression. The gene switch is inactivated by withdrawal of the SMR or, in the present application, virus-mediated lysis of the host cell. The present report presents the first study of immune responses induced by replication-competent controlled HSV-1 vectors in which one or two replication-essential genes are subjected to regulation by such a heat- and SMR-dependent gene switch.

(This article was submitted to an online preprint archive [32].)

## RESULTS

### Replication-competent controlled HSV-1 vectors.

The two-component HSP promoter-based gene switch employed in the vectors in the present study relied on the transactivator GLP65, which is activated by a narrow class of antiprogestins, including mifepristone and ulipristal (31, 33–35). GLP65 is a chimeric protein consisting of a DNA-binding domain from the Saccharomyces cerevisiae transcription factor GAL4, a truncated ligand-binding domain from a human progesterone receptor, and a transcriptional activation domain from human NF-κB protein P65. A DNA segment containing a promoter cassette consisting of a human HSP70B promoter and a GAL4-binding site-containing (GAL4-responsive) promoter and a functionally linked GLP65 gene was inserted between the UL43 and UL44 genes of the HSV-1 wild-type strain 17*syn*+. This intermediate vector was utilized for the construction of vectors HSV-GS3 and HSV-GS7. In vector HSV-GS3, the replication-essential immediate-early (IE) gene RS1/ICP4 and the early gene UL29/ICP8 had been placed under gene switch control by replacing the resident ICP4 and ICP8 promoters in the latter intermediate vector with GAL4-responsive promoters (Fig. 1B). In vector HSV-GS7, the replication-essential late gene UL38/VP19c had been subjected to gene switch control by replacement of its promoter with a GAL4-responsive promoter.

Replication of HSV-GS3 had previously been shown to be strictly dependent on activation by heat and antiprogestin in single-step growth experiments (35). When activated, the recombinant replicated nearly as efficiently as the wild-type virus, 17*syn*+. When administered to the footpads of mice, HSV-GS3 replicated only subsequent to an activating treatment with locally administered heat in the presence of systemic antiprogestin. The results of a single-step growth experiment with HSV-GS7 are shown in Fig. 1C. The recombinant replicated approximately as well as HSV-GS3 following heat treatment in the presence of the antiprogestin ulipristal (35). No replication was observed after either heat or antiprogestin treatment or in the absence of any treatment. To verify that HSV-GS7 replication was also tightly controlled *in vivo*, two of three groups of mice were administered HSV-GS7 virus (50,000 PFU per mouse) in the footpads, and the mice of one of the groups were given ulipristal intraperitoneally (i,p.) and, 3 h later, were subjected to a heat treatment to the footpads at 45°C for 10 min. One day later, all the mice were euthanized, and DNA and RNA were extracted from their feet and analyzed by quantitative PCR (qPCR) and RT-qPCR, respectively. Substantially larger amounts of HSV-1 DNA were detected in the feet of heat/ulipristal-treated mice than in untreated mice (Fig. 1D). Expression of several viral genes was observed for activated virus, but not for unactivated virus, strongly suggesting that viral replication and gene expression occurred only subsequent to heat/ulipristal activation.

### Protective immunity induced by activated HSV-GS3.

**FIG 2:**
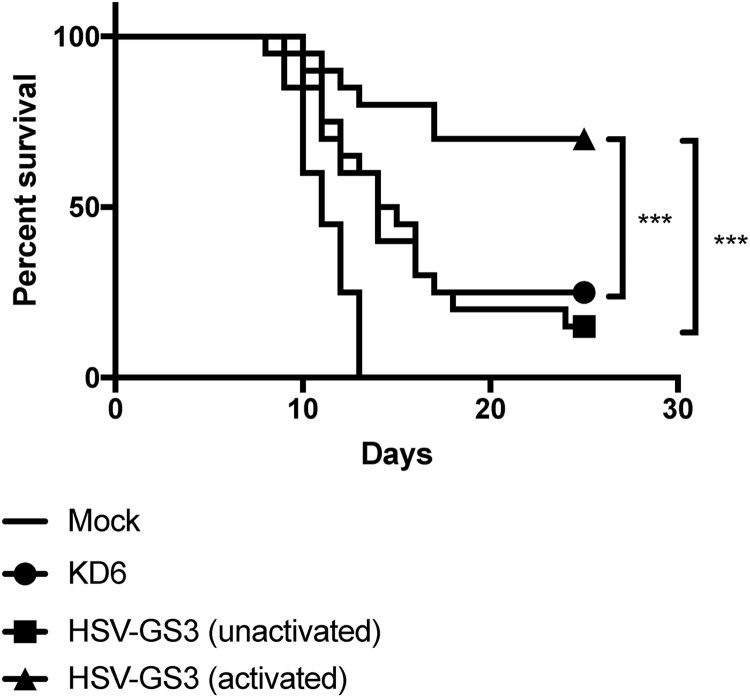
Immunization with HSV-GS3 protects against lethal challenge with HSV-1 17*syn*+ in the mouse footpad model. Groups of mice were inoculated on the plantar surfaces of both rear feet with 50,000 PFU/mouse of either the ICP4^−^ HSV-1 replication-incompetent recombinant KD6 or the HSV-1 replication-competent controlled recombinant HSV-GS3 (two groups) or were mock immunized with saline. The animals in one of the two HSV-GS3 groups received a 43.5°C/30-min heat treatment 3 h after virus inoculation in the systemic presence of mifepristone (0.5 mg/kg administered i.p. at the time of inoculation) [HSV-GS3 (activated)], whereas the animals in the other group were left untreated [HSV-GS3 (unactivated)]. At 22 days postimmunization, all the mice were challenged with a 20-fold lethal dose of wild-type HSV-1 strain 17*syn*+ applied to both rear feet. The data are presented as percent survival for each treatment group (*n* = 20 for each treatment). ***, *P* ≤ 0.05. See Materials and Methods for further details and Table 1 and Fig. 6 for information from comparable experiments.

Induction of protective immunity was evaluated in a mouse footpad lethal challenge model (36). In the first experiment, the virus vector HSV-GS3 or KD6 (an ICP4^−^ replication-incompetent HSV-1 recombinant [37]) was administered under anesthesia to the plantar surfaces of both rear feet of adult Swiss Webster outbred female mice (50,000 PFU per animal; 20 animals per group). Concurrently, and again 24 h later, the animals of one of the HSV-GS3 groups received an intraperitoneal injection of 0.5 mg/kg of body weight mifepristone. Three hours after inoculation, the mice in this group were subjected to heat treatment (43.5°C for 30 min) by immersion of their hind feet in a temperature-controlled water bath. Twenty-two days later, all the animals were challenged with a 20-fold lethal dose of the HSV-1 wild-type strain 17*syn*+ administered by the same route as the original virus inoculum. Survival of the animals was followed until no more lethal endpoints were reached, i.e., until all surviving animals had fully recovered (Fig. 2). KD6 induced a modest level of immunity. As had been expected, because it did not replicate and also did not express the major transcriptional regulator ICP4, unactivated HSV-GS3 provided a comparable degree of protection. Activated HSV-GS3 produced a far greater protective effect. Therefore, one round of efficient replication and/or expression of the master regulator ICP4, resulting in expression of all the viral proteins, produced a dramatic enhancement of protective immunity. It was noted that the heat and antiprogestin treatment had no systematic effect on the level of protection induced by the replication-defective virus KD6 (see “Statistical evaluation of data from all comparable experiments” below).

To determine whether immunization with activated HSV-GS3 reduced replication of the challenge virus more effectively than unactivated HSV-GS3 or the replication-defective KD6 virus, additional groups of animals (5 animals per group) were immunized and challenged with wild-type virus as described above. Four days after challenge, the animals were euthanized, their feet were dissected and homogenized, and virus present in the homogenates was titrated on rabbit skin (RS) cells. The results revealed that activated HSV-GS3 reduced challenge virus replication by nearly 2 orders of magnitude (6.2 × 10^3^ ± 1.5 × 10^2^ PFU) and unactivated recombinant virus (9.4 × 10^4^ ± 3.1 × 10^4^ PFU) or KD6 (4.0 × 10^4^ ± 8.3 × 10^2^ PFU) by only about 1 order of magnitude versus mock infection (5.5 × 10^5^ ± 1.2 × 10^4^ PFU).

**FIG 3:**
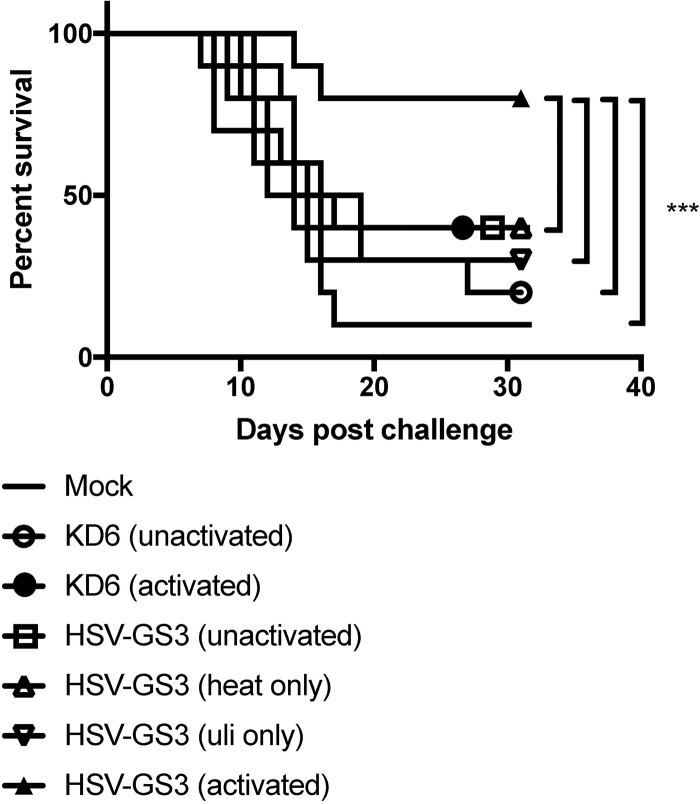
Gene switch-controlled immunization by recombinant HSV-GS3. Mice in four groups (*n* = 10 per group) were administered 500,000 PFU/mouse of HSV-GS3 on both rear feet. The first HSV-GS3 group received a 45°C/10-min heat treatment 3 h after inoculation in the systemic presence of ulipristal (50 μg/kg administered i.p. at the time of inoculation) [HSV-GS3 (activated)], the second group received only heat treatment [HSV-GS3 (heat only)], the third group was administered only ulipristal [HSV-GS3 (uli only)], and the fourth group was left untreated [HSV-GS3 (unactivated)]. A further group was mock immunized with saline. At 21 days, the animals in the HSV-GS3 groups were boosted with another 500,000-PFU/mouse dose of HSV-GS3, and 10 days later, all the mice were challenged with a 20-fold lethal dose of wild-type HSV-1 strain 17*syn*+ applied to both rear feet. The data are presented as percent survival for each treatment group. ***, *P* ≤ 0.05. See Materials and Methods for further details. Comparable data were obtained from a second experiment (not shown), in which mice were immunized with 50,000 PFU/mouse of HSV-GS3 and were not boosted.

**FIG 4:**
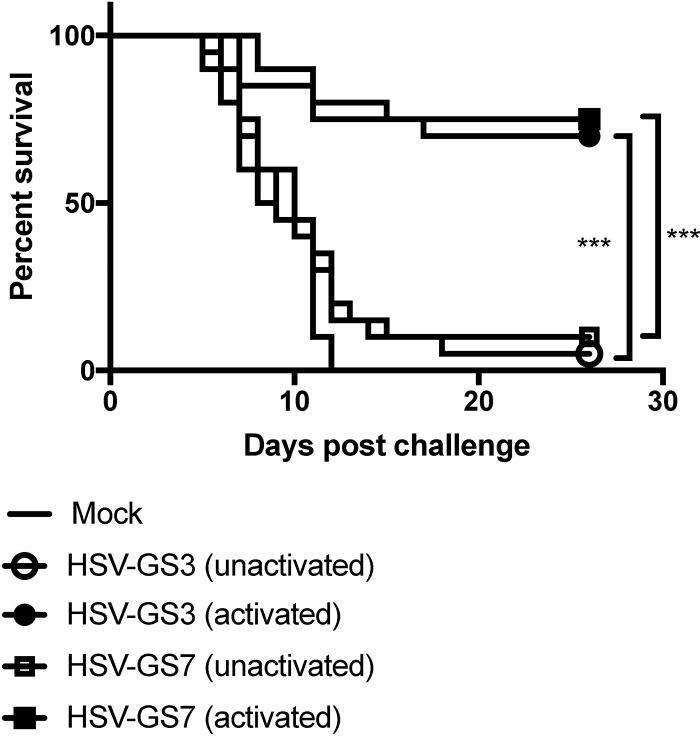
Immunization with HSV-1 replication-competent controlled recombinant HSV-GS7 and comparison with HSV-GS3. Mice were inoculated on both rear feet with 50,000 PFU/mouse of either HSV-GS3 or HSV-GS7 or were mock immunized with saline. For each recombinant, one group of animals was subjected to heat treatment in the presence of ulipristal as described in the legend to Fig. 3 (activated) and another group did not receive this treatment (unactivated). At 21 days postinoculation, all the mice were challenged with a 20-fold lethal dose of wild-type HSV-1 strain 17*syn*+ applied to both rear feet. The data are presented as percent survival for each treatment group (*n* = 20 for each HSV-GS3 or HSV-GS7 group; *n* = 10 for the mock-immunized group; ***, *P* ≤ 0.05). See Table 1 and Fig. 6 for information from comparable experiments.

**FIG 5:**
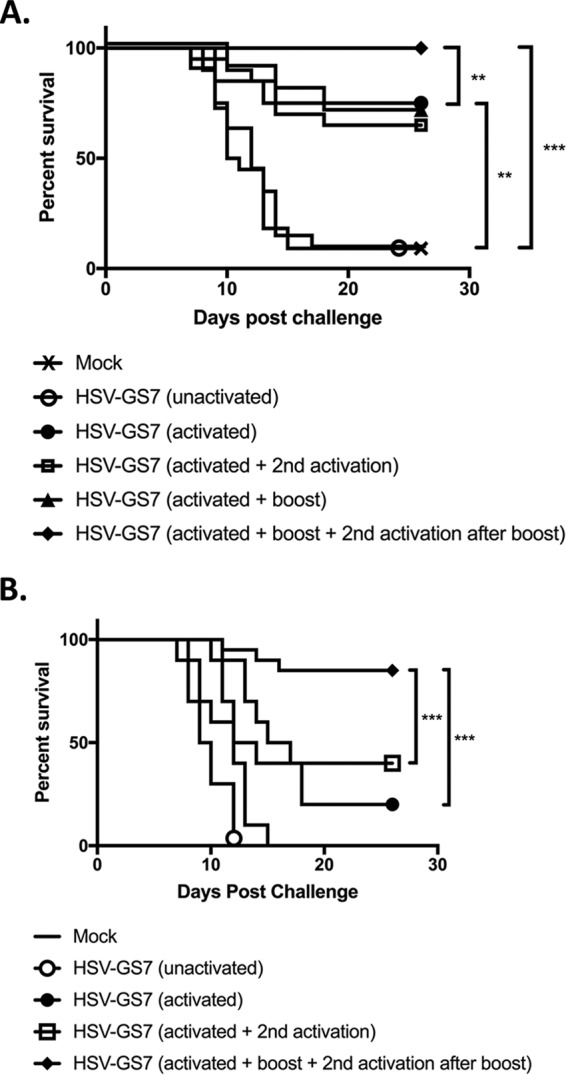
Effects of a second immunization or a second activation on HSV-GS7 efficacy. (A) Mice were inoculated on both rear feet with 50,000 PFU/mouse of HSV-GS7 or saline (mock; *n* = 10). HSV-GS7 groups were untreated (unactivated; *n* = 20); activated on day 1 (3 h after virus inoculation) by heat treatment at 45°C for 10 min in the presence of ulipristal (50 μg/kg administered i.p. at the time of inoculation) (activated; *n* = 20); activated on day 1 and reactivated 2 days later (activated + 2nd activation; *n* = 20); activated on day 1 and readministered 50,000 PFU/mouse of HSV-GS7 21 days later (activated + boost; *n* = 10); or activated on day 1, readministered 50,000 PFU/mouse of HSV-GS7 21 days after the first inoculation, and reactivated 3 h later (activated + boost + 2nd activation; *n* = 10). Twenty-one days after the last treatment, all the mice were challenged with a 20-fold lethal dose of wild-type HSV-1 strain 17*syn*+ applied to both rear feet. **, *P* ≤ 0.05; ***, *P* < 0.01. (B) Similar to panel A, except both initial and second immunizations were 5,000 PFU/mouse of HSV-GS7 (*n* = 10; for the second immunization, *n* = 20) (***, *P* ≤ 0.05). The data are presented as percent survival for each treatment group. See Table 1 and Fig. 6 for information on comparable experiments.

We next examined in a similar experiment whether induction of enhanced protective immunity was caused by activation of the heat- and antiprogestin-dependent gene switch that controls the expression of ICP4 and ICP8 in the HSV-GS3 recombinant. In this and all subsequent experiments, ulipristal was utilized instead of mifepristone because the former compound is a more active gene switch coactivator than the latter compound (35). Furthermore, the heat treatment regimen was changed to heat exposure at 45°C for 10 min to achieve greater reproducibility between experiments. The results revealed that enhanced protection was achieved only following activation of HSV-GS3 by heat treatment in the presence of ulipristal (Fig. 3). Heat treatment alone or ulipristal alone did not significantly increase the protective effect over that produced by the unactivated virus. These findings strongly suggested that it was the activation of the dual-responsive gene switch in HSV-GS3 that prompted the expression of the regulated replication-essential genes, as well as efficient virus replication that in turn resulted in the observed enhanced protective response. Note that in this experiment the virus inoculum was 500,000 PFU rather than 50,000 PFU, as in most other experiments (Table 1 and Fig. 2, 4, and 5A; see Fig. 7 and 8). This may account for the somewhat elevated level of protection induced by unactivated HSV-GS3 and KD6.

**TABLE 1:** Numbers and percentages of survivors in immunization/challenge experiments^*a*^

Virus	Dose (10^3^ PFU)	Activation	Second activation	Boost	Boost/activation	No. of mice in expt:								No./% of surviving mice
						1	2^*c*^	3	4	5^*d*^	6	7	8	
−		−	−	−	−	0 (10)	2 (10)	1 (10)	0 (10)	0 (10)	0 (10)	0 (10)	1 (10)	4 (80)/0
KD6	50	−	−	−	−	4 (10)	3 (10)		3 (10)					10 (30)/33
		+	−	−	−	2 (10)								2 (10)/20
	500	−						2 (10)						2 (10)/20
		+	−	−	−			4 (10)						4 (10)/40
HSV- GS3	50	−	−	−	−	3 (10)	4 (10)		3 (10)			1 (20)		11 (50)/22
		+	−	−	−	6 (10)	7 (10)		6 (10)			14 (20)		33 (50)/66
		+	+	−	−		8 (10)							8 (10)/80
	500	−	−	+	−			4 (10)						4 (10)/40
		+	−	+	−			8 (10)						8 (10)/80
		+	+	+	−			8 (10)						8 (10)/80
HSV-GS7	5	−	−	−	−					0 (10)		0 (10)	0 (10)	0 (30)/0
		+	−	−	−					5 (10)	5 (10)	2 (10)	4 (10)	16 (40)/40
		+	+	−	−						7 (10)	4 (10)		11 (20)/55
		+	−	−	+							17 (20)		17 (20)/85
		+	+	−	+						8 (10)	7 (10)		15 (20)/75
	50	−	−	−	−							2 (20)	2 (10)	4 (30)/13
		+	−	−	−						7 (10)	15 (20)	8 (10)	30 (40)/75
		+	+	−	−							13 (20)		13 (20)/65
		+	−	+	−						8 (10)	7 (10)		15 (20)/75
		+	−	+^*b*^	−						8 (10)			8 (10)/80
		+	−	−	+						10 (10)	10 (10)		20 (20)/100
		+	+	−	+							20 (20)		20 (20)/100
	500	−	−	−	−					6 (10)				6 (10)/60
		+	−	−	−					9 (10)				9 (10)/90

a Group sizes are shown in parentheses. Activation, regional heat treatment at 45°C for 10 min in the systemic presence of an anti-progestin (50 µg/kg ulipristal in most experiments.). Challenge, 10,000 PFU HSV-1 strain 17*syn*+. Boost/second immunization was with same virus dose that was used for the initial immunization. +, indicated treatment was administered; –, treatment was not administered.

b Boost with 500,000 PFU HSV-GS7.

c Challenge dose, 1,000 PFU.

d Activation at 44.5°C for 10 min. Data relating to incomplete activation (heat treatment only, or antiprogestin only) were omitted.

### HSV-GS3 and HSV-GS7 induce comparable protective responses.

The greatly enhanced protective response that resulted from transient activation of the gene switch in HSV-GS3-immunized animals could have been due to the efficient virus replication triggered by the activation and/or to the activation of the genes for the major transcription regulator ICP4, which in turn stimulated expression of all the other viral genes in primarily infected cells. Another question was related to whether abundant viral gene expression in secondarily infected cells (which may include antigen-presenting cells) could further enhance the immune response. Since the ICP4 genes are regulated in HSV-GS3, little expression of viral genes was expected to occur in the absence of another activating treatment. Recombinant HSV-GS7 in which the late VP19c gene had been subjected to regulation was constructed to examine these issues. HSV-GS3 and HSV-GS7 were compared in the mouse footpad challenge model. The results revealed that activated HSV-GS3 and HSV-GS7 provided similar levels of protection (Fig. 4). In the absence of activation, the protective effect of either recombinant was modest. Taking data from other experiments into account, the average protective effect for unactivated HSV-GS3 was 22% and that for unactivated HSV-GS7 was 13% (see “Statistical evaluation of data from all comparable experiments” below). The fact that the two recombinants that lacked expression of different proteins in the absence of activation induced comparable (modest) protective responses strongly suggested that it was not the absence of one or the other viral protein that limited the immune response to the recombinants. It was instead the vigorous replication of the activated recombinants that enhanced the protective responses against them. In secondarily infected cells (in which HSF1 was not activated), HSV-GS7 was expected to express all viral proteins except the regulated capsid protein at approximately normal levels. In contrast, HSV-GS3 all but lacked ICP4 expression in the absence of activation and exhibited dramatically curtailed expression of other viral proteins (35). The finding that HSV-GS3 and HSV-GS7 induced comparable protective responses suggested that viral proteins expressed in secondarily infected cells did not contribute in a relevant fashion to the immune response. However, we cannot exclude the possibility that the immune system was sufficiently sensitive to detect the very low levels of viral antigen expression occurring in cells secondarily infected with HSV-GS3.

### A second immunization further enhances protective immunity.

We next investigated whether a second activation treatment applied 2 days after the first treatment (i.e., at a time at which the initial round of replication of HSV-GS7 should have been completed) or a second immunization would further enhance protection against wild-type virus challenge. The second immunization was administered 3 weeks after the initial virus application. Booster virus was either activated or left unactivated. As shown in Fig. 5A, groups of mice were immunized with 50,000 PFU/animal of recombinant HSV-GS7. Activation of the virus induced an immune response that protected 75% of the animals after challenge with wild-type virus. After the second immunization with a similar dose of HSV-GS7, all the animals were protected. However, this occurred only if the booster virus was also subjected to an activating treatment. A second activation after a single dose of HSV-GS7 had no apparent effect. As effects of reimmunization may be more apparent in mice immunized with smaller quantities of virus, the experiment also featured parallel groups of animals that were immunized and boosted with 5,000 PFU/animal of recombinant HSV-GS7. As Fig. 5B documents, a second immunization followed by virus activation had an even more pronounced effect at the 10-fold-reduced virus doses. It was noted that in this part of the experiment a second activation appeared to modestly enhance the protective immune response (not statistically significant).

### Statistical evaluation of data from all comparable experiments.

**FIG 6:**
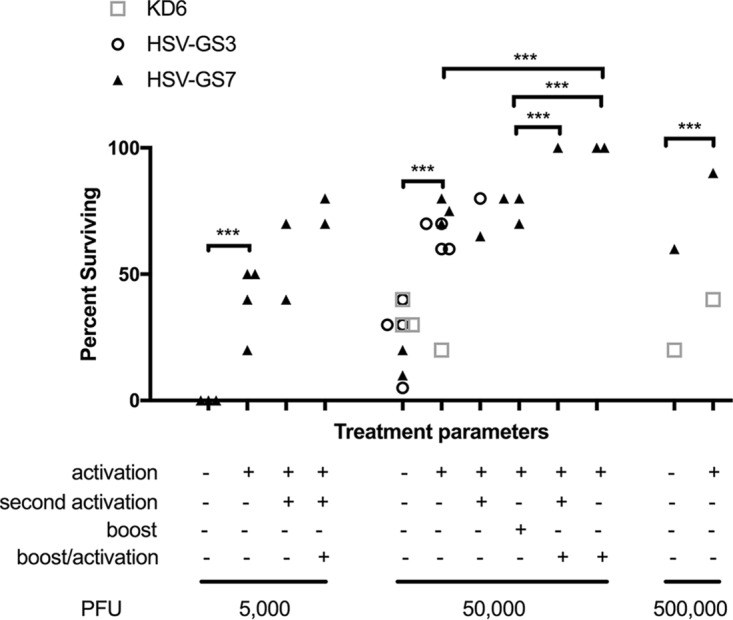
Meta-analysis of the efficacy of HSV-GS7 in protecting mice against lethal HSV-1 challenge across comparable experiments. Selected data from Table 1 showing percent survival for different treatment conditions examined for KD6, HSV-GS3, and/or HSV-GS7 are graphically depicted. The data sets for HSV-GS7 were analyzed using two-way ANOVA. ***, *P* ≤ 0.05.

Relevant data from all immunization/challenge experiments that were conducted using essentially identical protocols are assembled in Table 1. The results of a meta-analysis of the HSV-GS7 data are presented graphically in Fig. 6. The difference in immunization efficacy between activated and unactivated HSV-GS7 was found to be highly significant. Also significant was the increase in protection afforded by a second immunization with an activated HSV-GS7 vector. The effect of the second activation 2 days after virus administration and the first activation was not statistically relevant.

### Immune correlates of protection against challenge.

**FIG 7:**
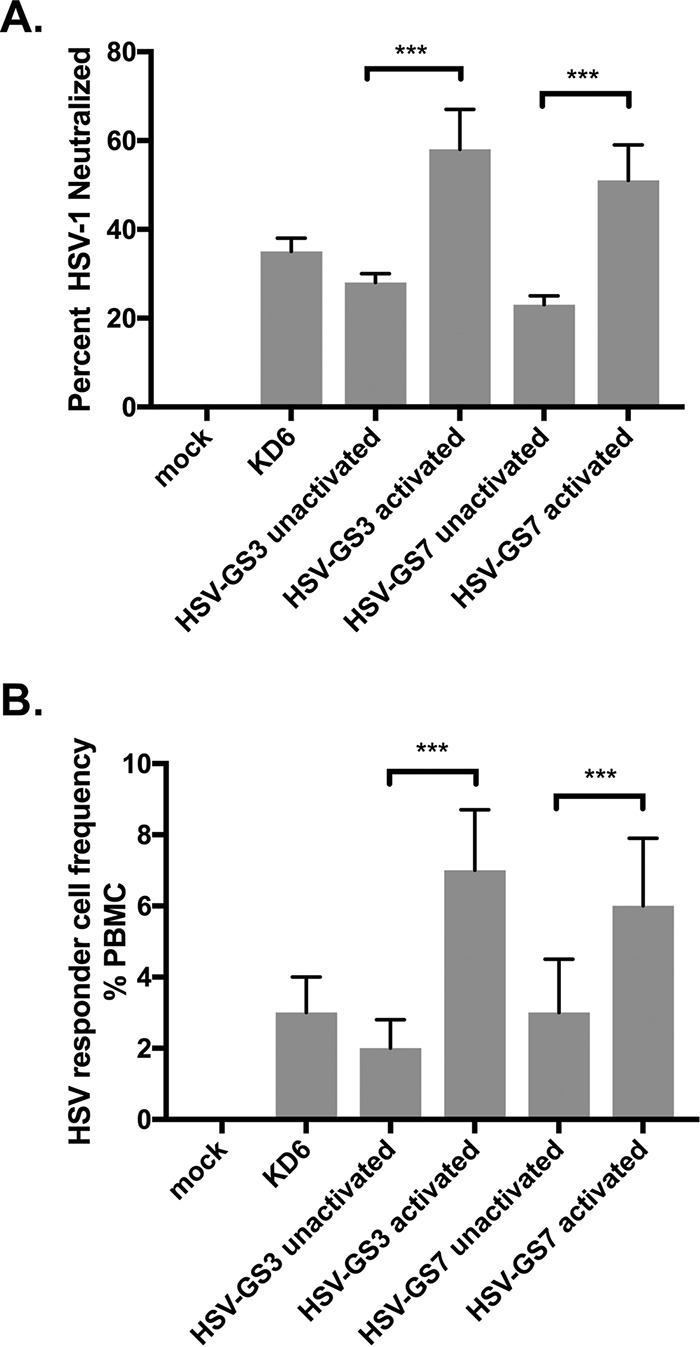
Anti-HSV-1 humoral and cellular immune responses. (A) Neutralizing antibodies induced by immunization. Dilutions of mouse serum samples (*n* = 5 for each experimental group) were tested for their ability to neutralize HSV-1, as described in Materials and Methods. The values are presented as averaged percentages of HSV-1 PFU neutralized for each group plus standard deviations. ANOVA; ***, *P* ≤ 0.05. See Materials and Methods for experimental details. (B) Responder cell frequency. The number of HSV-1-specific lymphocytes induced 3 weeks after immunization was determined by a limiting dilution lymphocyte proliferation assay. The data are presented as the averaged responder cell frequency for each experimental group plus standard deviation. Three blood/PBMC samples were analyzed per group. ANOVA; ***, *P* ≤ 0.05. See Materials and Methods for experimental details.

The presence and levels of antibodies capable of neutralizing HSV-1 strain 17*syn*+ were assessed in sera of mice that had been treated with activated or unactivated HSV-GS vectors or with the replication-incompetent virus KD6 (all at 50,000 PFU per animal) 3 weeks earlier. As expected, neutralizing antibodies could be detected subsequent to immunization with KD6 or unactivated HSV-GS (Fig. 7A). Antibody levels in sera from KD6-immunized and unactivated HSV-GS vector-immunized animals were comparable. Both activated HSV-GS3 and HSV-GS7 induced neutralizing antibody responses clearly superior to those induced by unactivated HSV-GS3 or HSV-GS7 or by KD6. To assess cellular immune responses, HSV-1-specific responder cells present in peripheral blood mononuclear cells (PBMCs) of mice that had been immunized 3 weeks earlier were quantified by a modified limiting dilution lymphoproliferation assay. Both unactivated HSV-GS vectors and KD6 virus were found to be capable of inducing detectable levels of HSV-1-specific responder cells (Fig. 7B). Substantially better responses were produced by activated HSV-GS3 and HSV-GS7.

### Immune responses to a heterologous antigen presented in the context of vigorous viral replication.

**FIG 8:**
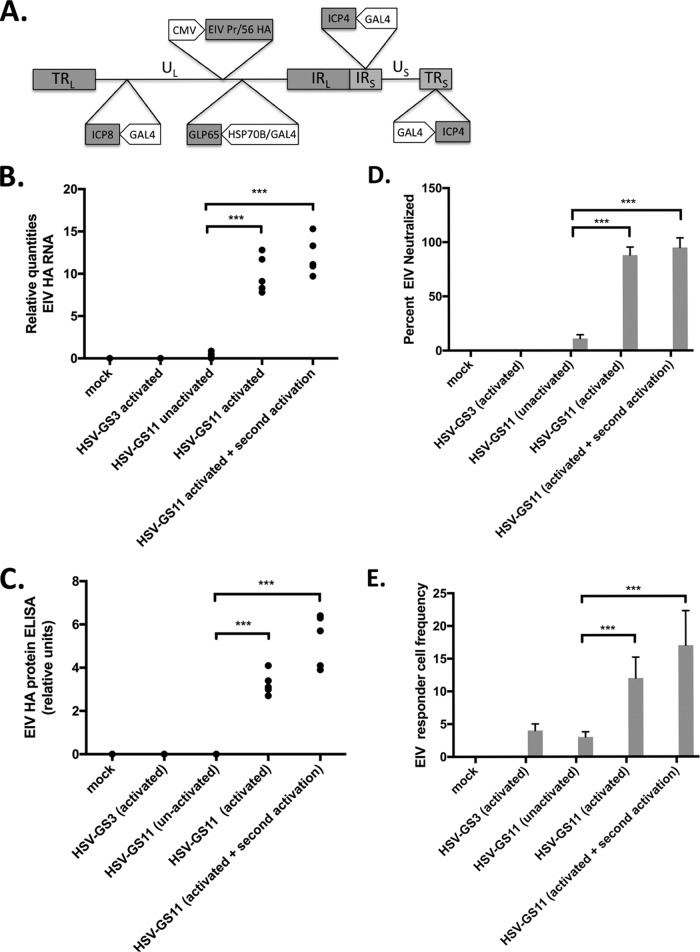
Immunization with activated HSV-GS11 induces a robust immune response against EIV Prague/56 HA. (A) HSV-GS11 vector containing the EIV Prague/56 (H7N7) HA gene driven by the CMV IE promoter and inserted into the intergenic region between UL37 and UL38. The HSP70/GAL4-GLP65 TA cassette was inserted into the intergenic region between UL43 and UL44. The promoters of the essential HSV-1 ICP4 and ICP8 genes were replaced with GAL4-responsive promoters. (B) Detection of EIV HA RNA expression following immunization of mice with HSV-GS11. Groups (*n* = 5) of 6- to 8-week old female BALB/c mice were inoculated on both rear footpads with either saline (mock), HSV-GS3 (50,000 PFU), or HSV-GS11 (50,000 PFU) as described in Materials and Methods. Vector replication was activated in some treatment groups by administration of heat and ulipristal as described in the legend to Fig. 3. One treatment group received a second activation treatment that was administered 2 days after the first activation treatment. Mouse feet were harvested 24 h after the last treatment, and RNA was isolated and cDNA prepared. Samples were analyzed by qPCR with EIV Prague/56 HA-specific TaqMan primers/probe (Table 2). Normalization of RNA quantities was relative to the APRT cellular gene. ANOVA; ***, *P* ≤ 0.05. (C) Detection of EIV HA antigen by ELISA following immunization of mice with HSV-GS11. Groups (*n* = 5) of 6- to 8-week old female BALB/c mice were inoculated on both rear footpads with either saline (mock), HSV-GS3 (50,000 PFU), or HSV-GS11 (50,000 PFU) and subjected to the same treatments as for panel B. Mouse feet were harvested 24 h after the last treatment, and protein homogenates were analyzed using an EIV Prague/56 HA-specific ELISA as described in Materials and Methods. The data are presented as *S*/*N* ratios. ANOVA; ***, *P* ≤ 0.05. (D) Neutralizing antibodies induced by immunization. Groups (*n* = 5) of 6- to 8-week old female BALB/c mice were administered HSV-GS3, HSV-GS7, or saline (mock) and treated as for panel B. Twenty-one days postimmunization, mouse serum samples were tested for their ability to neutralize EIV Prague/56. The values are presented as percentages of EIV Prague/56 HA PFU neutralized. (E) Responder cell frequency assay. Immunizations and treatments were as for panel D. Twenty-one days postimmunization, the number of EIV Prague/56 HA-specific lymphocytes was determined by a limiting dilution lymphocyte proliferation assay. The data are presented as the responder cell frequency of each experimental group.

To evaluate immune responses to a heterologous antigen expressed from an HSV-GS vector, recombinant HSV-GS11 was constructed. The recombinant was derived from HSV-GS3 by the insertion in the UL37-UL38 intergenic region of an expression cassette comprising a gene for an equine influenza virus (EIV) hemagglutinin (HA) (EIV Prague/56 HA) functionally linked to a cytomegalovirus (CMV) (immediate-early) promoter (Fig. 8A). Expression of the influenza virus gene was verified in groups of adult female BALB/c mice inoculated on the rear footpads with either saline, HSV-GS3, or HSV-GS11 (50,000 PFU per animal). The HSV-GS3 group, as well as one of the HSV-GS11 groups, was subjected to activation treatment. Another HSV-GS11 group underwent an activation treatment twice; the second treatment was administered 2 days after the first treatment. One day after the last treatment, the animals were euthanized, and RNA was extracted from one hind foot and protein from the other. The results from an RT-qPCR analysis of HA gene expression are shown in Fig. 8B and the results from an EIV Prague/56 HA-specific enzyme-linked immunosorbent assay (ELISA) in Fig. 8C. HA RNA and HA could be detected in samples from HSV-GS11-inoculated animals, most abundantly when the animals had been subjected to activation treatment. Both HA RNA and protein levels appeared to be somewhat higher in twice-activated animals than in once-activated animals.

To assess immune responses, additional groups of mice were inoculated with saline (mock immunization) or with 50,000 PFU of HSV-GS3 or HSV-GS11 (three groups). All the animals in the HSV-GS3 group and in two of the HSV-GS11 groups were subjected to an activation treatment. The animals of one of the HSV-GS11 groups received a second activation treatment 2 days later. All the animals were sacrificed 21 days postimmunization. Serum samples were tested for their ability to neutralize EIV Prague/56. As expected, neutralizing antibodies were not detected in unimmunized (not shown), mock-immunized, or vector-immunized animals (Fig. 8D). Unactivated HSV-GS11 was capable of inducing a modest neutralizing antibody response. Activation of HSV-GS11 shortly after inoculation resulted in a severalfold-magnified response. It was noted that twice-activated HSV-GS11 elicited a response only marginally better than that elicited by once-activated virus (not statistically significant). HA-specific responder cells present in PBMCs were quantified by the same type of responder cell frequency (RCF) assay that had been used to assess the numbers of HSV-1-specific responder cells. HA-specific responder cells were not detected in unimmunized (not shown) or mock-immunized (Fig. 8E) animals. Induction of a cellular immune response was observed in animals immunized with HSV-GS11 but not subjected to an activation treatment. An activated vector (HSV-GS3) produced a similar response. Far greater numbers of HA-specific responder cells were found in animals immunized with once- or twice-activated HSV-GS11.

## DISCUSSION

The once-activated replication-competent controlled HSV-1 recombinants HSV-GS3 and HSV-GS7 induced considerably more potent protective immune responses than the replication-defective HSV-1 strain KD6. Twice-immunized animals were completely protected against lethal challenge. The levels of partial protection engendered by unactivated HSV-GS3, unactivated HSV-GS7, and KD6 (lacking expression of different viral proteins) were comparable, which strongly suggests that the much-enhanced protection provided by the activated HSV-GS recombinants was a consequence of their ability to efficiently replicate. Both better neutralizing antibody and better cellular immune responses were induced by the activated HSV-GS vectors, indicating that the replicating vectors not only boosted cellular immunity, but broadly enhanced immune responses. Generalizing these findings, it appears that replication-competent controlled vectors are promising new agents that may better protect against diseases caused by the viruses from which they were derived than replication-incompetent/inactivated vaccines and possibly also live attenuated vaccines. Perhaps even more important, our observations suggest that replication-competent controlled vectors can be excellent immunization platforms that may be exploited for the elaboration of new “vaccines.” Expression of an influenza virus antigen induced far more potent neutralizing antibody and antigen-specific responder cell responses when the antigen was expressed in the context of vector replication than in the absence of vector replication. To the extent that such a comparison is valid, neutralizing antibody responses, as well as antigen-specific responder cell responses, against the influenza virus antigen elicited by activated HSV-GS11 were both stronger than the corresponding responses against vector antigens.

A second activation treatment administered 2 days after the initial activation of a replication-competent controlled HSV-1 vector did not consistently enhance the protective immune response against challenge by the wild-type virus from which the vector was derived. Also, humoral and cellular immune responses against an influenza virus antigen expressed from a replication-competent controlled HSV-1 vector were only marginally enhanced by a second activation treatment. At this time, we cannot determine whether the second activation was only marginally effective because the immune system was already maximally engaged by the initial immunization or because optimal conditions for the second activation treatment had not been identified.

Immunization by replication-competent controlled vectors represents a novel paradigm that may be elaborated in various ways. Therefore, the work presented here should be regarded as a first illustration of the principle. In general, any of a number of DNA viruses, including members of all the herpesvirus subfamilies, may be employed for the construction of replication-competent controlled vectors. We selected a fully virulent HSV-1 strain as the backbone of the GS vectors for several reasons, including the fact that HSV has a double-stranded DNA genome that does not integrate into the host genome and that it is tolerant of sizeable insertions, supports stable expression of heterologous genes, replicates and lyses cells efficiently, and responds to antiviral drugs (38–41). Although HSV is endemic in the human population, available data indicate that preexisting immunity is not an obstacle to vaccination/immunization uses of HSV vectors (discussed in reference 35). Furthermore, latent infection was considered a manageable issue because replication of replication-competent controlled vectors can be stringently controlled, and reactivation should not be capable of occurring in the absence of activation.

Regarding the two-component HSP promoter-based gene switch that is used for controlling viral replication, several ligand-dependent transactivators are known that may be considered for incorporation in such a gene switch. They include transactivators that are derived from the bacterial tetracycline repressor or other bacterial repressors, as well as chimeric transactivators that exploit ligand-binding domains of various steroid receptors, e.g., human progesterone, human estrogen, or insect ecdysone receptors, or are activated by dimerization (28). We previously constructed and analyzed two-component HSP promoter-based gene switches that are coactivated by antiprogestins, ecdysteroid ponasterone A, and rapamycin/rapalog (31, 42). For regulating the HSV-GS vectors, we made use of a heat- and antiprogestin-activated gene switch that is strictly dependent on both heat and antiprogestin. While it has been more thoroughly characterized than any other (31), this gene switch also recommends itself for several other reasons. It comprises the transactivator GLP65, which is activated by a narrow spectrum of antiprogestins but not progestins (35). Since it includes only a truncated ligand-binding domain of a progesterone receptor, GLP65 should not be susceptible to ligand-independent activation; in fact, after many years of experimental use, no antiprogestin-independent activation has been reported. At the low doses at which they activate GLP65, antiprogestins, such as mifepristone and ulipristal, are not expected to have any negative effects on the immune system (26). These antiprogestins have also been safety tested in human subjects and are readily available. They are not considered essential active ingredients by the WHO, and their primary use has been in emergency contraception. To maintain maximal safety in a human immunization scenario, i.e., to preclude any possibility of reactivation from latency, antiprogestins should not be administered subsequent to immunization with an HSV-GS-type vector. Therefore, the vectors may not be employed for immunization of healthy female subjects of reproductive age or younger, or the subjects may forego later use of antiprogestins for emergency contraception. In contrast, the HSV-GS vectors may be well suited to be immunization agents or platforms in middle-aged or elderly subjects. Note that it may be feasible to disable replication of fully activated HSV-GS vectors in latently infected cells through further engineering.

Replication-competent controlled virus vectors may be capable of making a unique contribution to immunization of immunocompromised patients. It has been estimated that in 2012 over 3% of the adult populations of the United States and the United Kingdom were immunocompromised (43). These patients are particularly vulnerable to vaccine-preventable disease and would greatly benefit from effective vaccination. Owing to their condition, their immune response to inactivated or subunit vaccines may be inadequate. Unfortunately, the use of live attenuated vaccines that might produce a more balanced and more persistent immune response is problematic because of the perceived danger that such vaccines may be insufficiently controlled by the weakened immune system and may cause the disease they are intended to prevent. According to most relevant guidelines, live attenuated vaccines are contraindicated in patients under immunosuppressive therapy or in HIV patients with low CD4 counts (44). Hence, use of currently marketed vaccines against tuberculosis (BCG); measles, mumps, and rubella; varicella-zoster virus; rotavirus; influenza virus (nasal); and yellow fever virus is universally discouraged. Newer, more effective vaccines under development, e.g., the new pertussis vaccine BPZE1 (45), also may not become available to the immunocompromised. Replication-competent controlled virus vectors expressing antigens of these microbes in the context of efficient viral replication can be expected to elicit immune reactions similar to or more potent than those of the contraindicated live attenuated vaccines. Escape from immune control will not be an issue because replication of the vectors can be activated and safely deactivated independent of the immune status of a patient.

In this report, we have focused on the immunization effects of once-activated replication-competent controlled vectors that undergo one round of replication. We note that viral vectors that were intended to be limited to one cycle of replication were advanced before as potential vaccines (46). These so-called disabled infectious single-cycle (DISC) viruses typically had the gene for glycoprotein H, which is critical for infection, deleted. Comparing DISC viruses and replication-competent controlled viruses, the latter replicate nearly as efficiently as the wild-type virus that was used for their construction while no comparable information is available for the DISC viruses that were advanced as vaccine candidates. Replication of DISC viruses is not localized but occurs throughout the host. Depending on the dose of virus administered, a single round of replication of disseminated virus can be expected to result in significant toxicity. Not surprisingly, intracranial administration of the DISC virus TA-HSV resulted in neurotoxicity that was only about 2 orders of magnitude lower than that of the wild-type virus from which the strain was derived (47). Replication-competent controlled viruses should be devoid of such toxicity because they are virtually incapable of replicating unless deliberately activated. We note that the stringency of the replication block could be increased further by subjecting additional replication-essential genes to gene switch control. Furthermore, even though progeny virus produced by a DISC virus upon immunization is not supposed to be capable of reinfecting cells, latent infection has been observed (47). Latent infection is known to involve spread from cell to cell (48). Hence, as has been pointed out before (49), DISC viruses, such as TA-HSV, appear to be leaky for replication *in vivo*. The above-mentioned properties of DISC viruses are not desirable in an immunization agent or vector administered to healthy people and would disqualify such viruses from use in persons with impaired immune systems. A clinical trial was conducted that investigated a DISC virus as a therapeutic vaccine against recurrent genital herpes (50). The results were negative. The DISC virus (TA-HSV) studied had been derived from a minimally virulent HSV-2 wild-type strain (47, 51). Moreover, the virus was administered at a relatively low dose, apparently to avoid toxicity. The failure of the trial should not be surprising.

Unlike a conventional vaccine, a replication-competent controlled vector requires activation after administration to a subject to be immunized. Activation will need to be neither costly nor inconvenient. A single ulipristal dose can be administered orally at the time of immunization. Regarding heat treatment, we have developed small pads that conduction heat the inoculation region by crystallization of a supercooled salt solution (52). The pads are capable of maintaining a temperature of 45°C for 15 min. This heat dose was shown to be sufficient for strong activation of the HSP70B promoter, which is the promoter used in the two-component gene switch that controls the HSV-GS vectors, in all layers of the skin of human subjects.

## MATERIALS AND METHODS

### Cells, plasmids, and viruses.

RS cells were a gift from E. Wagner and were propagated in minimal essential medium with Eagle's salts (MEM) (Life Technologies/Thermo Fisher Scientific) supplemented with 5% heat-inactivated calf serum (Atlanta Biologicals, Lawrenceville, GA), 292 mg/ml l-glutamine, 250 U/ml of penicillin, and 250 μg/ml of streptomycin (Life Technologies). Vero cells (procured from the American Type Culture Collection [ATCC]) and Vero cell-derived E5 cells (53) (obtained from N. DeLuca) were propagated in Dulbecco's modified Eagle's medium (DMEM) with 10% heat-inactivated fetal calf serum, 250 U/ml of penicillin, and 250 μg/ml of streptomycin. HSV-1 strain 17*syn*+ and ICP4 deletion strain KD6 (37) were obtained from J. Stevens. Stocks of KD6, HSV-GS3, and HSV-GS7 were propagated on E5 cells. For HSV-GS3 and HSV-GS7, the medium was supplemented with 10 nM mifepristone, and infected cultures were subjected to daily heat treatment at 43.5°C for 30 min for three successive days. EIV Prague/56 was a gift from T. Lengsfeld and was propagated on Madin-Darby canine kidney (MDCK) cells (ATCC) in DMEM supplemented with 10% heat-inactivated horse serum as previously described (54). Stocks of EIV were titrated using 50% tissue culture infective dose (TCID_50_) assays in MDCK cells. All the cells were cultured at 37°C under 5% CO_2_.

### Chemical reagents.

Mifepristone was obtained from Sigma-Aldrich, and ulipristal acetate was from D-Innovation Pharmaceutical Inc., Chengdu, Sichuan, People's Republic of China. Both compounds were USP grade.

### Construction of viruses.

All the viruses were constructed using wild-type HSV-1 strain 17*syn*+ as the backbone. Viral recombinants were generated by homologous recombination of engineered plasmids, along with purified virion DNA, in RS cells transfected by the calcium phosphate precipitation method, as previously described (41). Plasmids used to engineer the insertions of the transactivator cassette or the GAL4-responsive promoters comprised HSV-1 sequences cloned from strain 17*syn*+. HSV-GS3 (Fig. 1B) was constructed as previously described (3, 35). HSV-GS7 was derived from the intermediate vector HSV-17GS43 (35), which contains an insertion between the UL43 and UL44 genes of a transactivator cassette containing a GLP65 gene under the control of a promoter cassette that combined a human HSP70B promoter and a GAL4-responsive promoter (31). To place the UL38/VP19c gene under the regulation of a GAL4-responsive promoter, plasmid pBS-KS:GAL4-UL38 was constructed. The plasmid contains a GAL4 promoter inserted between the HSV-1 UL38 recombination arms of the plasmid pBS-KS:UL38Δpromoter. A GAL4-responsive promoter comprising six copies of the yeast GAL4 upstream activating sequence (UAS), the adenovirus E1b TATA sequence, and the synthetic intron Ivs8 was excised from the plasmid pGene/V5-His A (Invitrogen, Life Technologies) with AatII and HindIII, and the resulting 473-bp fragment was gel purified. For the vector, pBS-KS:UL38Δpromoter was digested with AatII and HindIII, and the resulting 4,285-bp fragment was gel purified and treated with shrimp alkaline phosphatase. Ligation of the last two fragments placed the GAL4 promoter in front of the UL38 transcriptional start site. To produce recombinant HSV-GS7, RS cells were cotransfected with the plasmid pBS-KS:GAL4-UL38 and purified HSV-17GS43 virion DNA. Subsequent to the addition of mifepristone to the medium, the cotransfected cells were exposed to 43.5°C for 30 min and then incubated at 37°C. Subsequently, on days 2 and 3, the cells were again incubated at 43.5°C for 30 min and then returned to 37°C. Picking and amplification of plaques, screening, and plaque purification were performed essentially as described for HSV-GS3 (3, 35). The resulting plaque-purified HSV-GS7 was verified by Southern blotting, as well as by PCR and DNA sequence analysis of the recombination junctions. Plasmid pBS-KS:UL38Δpromoter was constructed by deletion of the region from −1 to −47 of the UL38 promoter, i.e., by synthesizing two PCR fragments (one 437 bp and the other 550 bp) on either side of the deletion and cloning them into pBS KS+. HSV-GS11 was derived from the vector HSV-GS3 (35) and contains an insertion between the UL37 and UL38 genes of a gene cassette expressing the EIV Prague/56 HA gene driven by the CMV IE promoter. A recombination plasmid was constructed by the following sequential steps. First, an 814-bp fragment containing the region spanning the HSV-1 UL37/UL38 intergenic region from nucleotides (nt) 83603 to 84417 from plasmid NK470 was subcloned into pBS that had had the multiple-cloning site (MCS) removed (by digestion with KpnI/SacI) to yield pBS:UL37/38. A cassette containing a synthetic CMV IE promoter flanked by the pBS-SK+ MCS was ligated into pBS:UL37/38 digested with BspE1/AflII, which cut between the UL37 and UL38 genes, to yield the plasmid pIN:UL37/38. The EIV Prague/56 HA gene was PCR cloned from cDNA prepared from EIV Prague/56. Briefly, RNA was prepared by TRIzol extraction of a stock of EIV Prague 56 and was reverse transcribed using Omni-Script reverse transcriptase (Qiagen) according to the manufacturer's instructions. The cDNAs were cloned into pBS, and the clone containing the HA gene (pBS-EIVPrague56/HA) was confirmed by sequence analysis. The Prague/56 HA gene was subcloned from this plasmid and inserted behind the CMV promoter in the plasmid pIN:UL37/38 to yield plasmid pIN:37/38-Prague56/HA. To produce recombinant HSV-GS11, RS cells were cotransfected with plasmid pIN:37/38-Prague56/HA and purified HSV-GS3 virion DNA. Subsequent to the addition of mifepristone to the medium, the cotransfected cells were exposed to 43.5°C for 30 min and then incubated at 37°C. Subsequently, on days 2 and 3, the cells were again incubated at 43.5°C for 30 min and then returned to 37°C. Picking and amplification of plaques, screening, and plaque purification were performed essentially as described for HSV-GS3 (3, 35). The resulting plaque-purified HSV-GS11 was verified by Southern blotting, as well as by PCR and DNA sequence analysis of the recombination junctions.

### Construction of expression plasmid pVP19c.

Plasmid pVP19c was constructed by PCR cloning of the promoter and coding sequence of the HSV-1 VP19c gene into pBS KS+ using HSV-1 strain 17*syn*+ virion DNA as the PCR target.

### Single-step growth analysis.

Confluent monolayers of Vero cells were infected with HSV-GS7 at a multiplicity of infection (MOI) of 3. Virus was allowed to adsorb for 1 h at 37°C; then, the inoculum was removed, and the cells were overlaid with complete medium. Ulipristal treatment (10 nM) was initiated at the time of the initial infection. Heat treatment was performed either immediately after infection or 4 h later by floating the sealed dishes in a 43.5°C water bath for 30 min. The dishes were then incubated for 72 h at 37°C. At 0, 4 or 8, 12 or 16, and 24 or 28 h postinfection, two dishes were removed, and the cells were scraped into medium for harvesting and subjected to two freeze-thaw cycles. Infectious virus was then determined by titrating the lysate of each dish in triplicate on 24-well plates of confluent E5 cells transfected 24 h prior to infection with the expression vector pVP19c using Lipofectamine 2000 (Life Technologies). Plaques were visualized 2 days after infection using an antibody plaque assay, essentially as previously described (35).

### Immunization.

Briefly, 4- to 6-week-old female outbred Swiss Webster mice (Envigo, Tampa, FL) (or BALB/c mice [Envigo, Tampa, FL] for experiments involving HSV-GS11) were inoculated with the appropriate viral vector or phosphate-buffered saline (PBS) (pH 7.3; 50 μl/mouse) on the lightly abraded plantar surfaces of both rear feet as previously described (41). To facilitate efficient uptake of the vector, and to minimize the amount of abrasion required, the feet were saline treated prior to infection. The following procedure was employed. Mice were first anesthetized with isoflurane by inhalation. Flunixin meglumine (1.1 mg/kg) was then administered intramuscularly (i.m.) to alleviate any pain associated with the procedure. Thereafter, 25 to 50 μl (no more than 50 μl) of sterile 10% saline was injected subepidermally under both rear footpads. The mice were then returned to their cages. Four hours later, the mice were anesthetized by i.m. injection of 10 to 20 μl of a cocktail of acepromazine (2.5 to 3.75 mg/kg), xylazine (7.5 to 11.5 mg/kg), and ketamine (30 to 45 mg/kg). Both rear footpads of the anesthetized mice were then lightly abraded with an emery board to scratch the keratinized layer of the skin to allow the virus to adsorb efficiently. The anesthetized mice were rested on their backs, and 50 μl of the appropriate dilution of viral vector was placed on the footpads using a pipette. The viral vector was allowed to adsorb until the mice awoke. To activate vector replication, a combination of heat and mifepristone/ulipristal treatment was used. Heat treatment was performed by immersion of the hind limbs in a temperature-controlled water bath 3 h after virus administration. The mice were allowed to recover at 37°C for 15 min. Mifepristone (0.5 mg/kg) or ulipristal (50 μg/kg) in dimethyl sulfoxide (DMSO) was administered intraperitoneally (i.p.) at the time of immunization and again 24 h later.

### HSV-1 challenge in mice.

Immunized and control mice were challenged by infection on both rear footpads with 10,000 PFU, typically, of HSV-1 strain 17*syn*+. The procedure for the saline pretreatment, anesthesia, and application of virus was the same as that described above for application of the viral vector, except that no mifepristone/ulipristal or heat treatment was administered. For the efficacy determination, a modified endpoint analysis was used. The mice were monitored daily (with cages coded in a masked fashion). When the mice reached clinical endpoints indicating severe central nervous system (CNS) infection (bilateral hind limb paralysis, inability to move when touched, or trembling), they were euthanized.

### Blood collection, and serum and lymphocyte isolation.

For serum neutralization studies, blood was collected by retro-orbital bleeding prior to immunization. For retro-orbital bleeding, mice were anesthetized with ketamine (90 mg/kg) and xylazine (10 mg/kg) i.p. For the collection of serum and lymphocytes at 3 weeks after immunization, mice from each group were anesthetized by inhalation of 2 to 3% isoflurane. The total blood volume of each mouse was collected, and the mice were euthanized by cervical dislocation. PBMCs were isolated by Ficoll gradient separation using Lymphoprep (Miltenyi Biotec, Bergisch Gladbach. Germany) according to the manufacturer's protocol.

### Neutralizing antibody assay.

After collection, the blood was allowed to clot for 30 min. After centrifugation at 800 × *g*, the serum was collected. Serum samples were heated to 56°C for 1 h to inactivate complement and were then diluted 1:10 in complete DMEM containing 10% heat-inactivated fetal bovine serum (FBS). For HSV-1 neutralization, 50 μl of a suspension containing approximately 100 PFU of HSV-1 strain 17*syn*+ was added to each dilution of serum to a final volume of 100 μl. For EIV neutralization, 50 μl of a suspension containing approximately 100 TCID_50_ units of EIV Prague/56 were added to each dilution of serum to a final volume of 100 μl. The initial serum dilution, therefore, was 1:20. The serum-virus mixtures were placed on a rocker at room temperature for 1 h, and the amount of virus that was not neutralized at a given concentration of serum was titrated on RS (HSV-1) or MDCK (EIV) cell monolayers in order to calculate the neutralizing antibody titers.

### RCF assay.

HSV-1- or EIV-specific responder cells were quantified by a modified limiting dilution lymphoproliferation assay (55), using either cell-free HSV-1 or EIV Prague/56 HA protein lysate and control antigen lysate. Briefly, wells of 96-well plates were coated with 20 μl/well of antigen extract (HSV-1 or RS cell control lysate, or EIV Prague/56 HA or Vero cell control lysate) and were allowed to air dry in a laminar flow hood. Dilutions of mouse PBMCs in DMEM were added to each well so that each well contained a minimum of 1 and a maximum of 10 lymphocytes per well in a volume of 100 μl complete medium (with serum). The plates were then incubated at 37°C. After 24 h, medium containing 10 μCi of [^3^H]thymidine was added to each plate for 12 h, the medium was replaced, and the plates were incubated for an additional 72 h. The wells were harvested, and the DNA was precipitated in 20 volumes of cold 10% trichloroacetic acid, transferred onto glass fiber discs (Whatman; GF/C) by filtration, rinsed with 95% ethanol, and dried using a heat lamp. The filters were then transferred to scintillation vials with Scintiverse (Fisher Scientific) and counted. The counts per minute (cpm) of [^3^H]thymidine were converted to RCF using the maximum-likelihood estimate method of Levin et al. (56).

### Viral DNA replication and transcription.

Groups (*n* = 5) of 4- to 6-week-old female outbred Swiss Webster mice were anesthetized and inoculated with an HSV-GS vector on both rear footpads as described above. Ulipristal (50 μg/kg) was administered i.p. at the time of infection. Three hours after virus administration, heat treatment was performed by immersion of the rear feet in a 45°C water bath for 10 min. The mice were allowed to recover at 37°C for 15 min. The mice were sacrificed 24 h after heat induction, and the feet were dissected and snap-frozen in RNAlater (Sigma-Aldrich). DNA and RNA were extracted by grinding the tissues in TRIzol (Thermo Fisher Scientific) and back extracting the DNA from the interface. The extracted DNA was subjected to TaqMan real-time PCR (qPCR) for quantitative analysis of HSV-1 DNA (using HSV-1 DNA polymerase primers/probe) (57). RNA was analyzed by reverse transcription and TaqMan real-time PCR (RT-qPCR) for the presence of ICP4, thymidine kinase (TK), capsid protein VP19c, and glycoprotein C (gC) transcripts. Normalization of DNA and RNA quantities was relative to the adenine phosphoribosyltransferase (APRT) cellular gene. The sequences of the primers and probes used are provided in Table 2.

**TABLE 2:** Primers and probes used for qPCR

Target	Orientation/probe^*a*^	Sequence (5′–3′)
HSV DNA Pol	F	AGAGGGACATCCAGGACTTTGT
	R	CAGGCGCTTGTTGGTGTAC
	P	ACCGCCGAACTGAGCA
ICP4	F	CACGGGCCGCTTCAC
	R	GCGATAGCGCGCGTAGA
	P	CCGACGCGACCTCC
gC	F	CCTCCACGCCCAAAAGC
	R	GGTGGTGTTGTTCTTGGGTTTG
	P	CCCCACGTCCACCCC
TK	F	CACGCTACTGCGGGTTTATATAGAC
	R	GGCTCGGGTACGTAGACGATAT
	P	CACCACGCAACTGC
VP19c	F	CTTGGTCGACGAGCTGTTTG
	R	GCCCCGTTGCGTACAG
	P	CCGTCCGCGTTCATG
EIV Prague/56 HA	F	GGAACATTAGAATTCACAGCAGAGG
	R	CCTGTTCTCAATTTAACATATCCCC
	P	GGGGATATGTTAAAT
Mouse APRT	F	CTCAAGAAATCTAACCCCTGACTCA
	R	GCGGGACAGGCTGAGA
	P	CCCCACACACACCTC

a F, forward; R, reverse; P, probe.

### EIV Prague/56 HA ELISA.

In order to produce the antigen for the ELISA, the insert from plasmid pBS-EIVPrague56/HA (described above) was subcloned into the expression vector pET-31b (Millipore), and the EIVPrague56/HA protein was expressed in Escherichia coli in accordance with the manufacturer's instructions. After induction and growth, the bacteria were lysed, proteins were isolated in the presence of protease inhibitors, and the proteins were used to coat a 96-well ELISA plate, which was allowed to air dry. Dilutions (1:10 to 1:200) of murine serum were applied to the wells, and the plate was incubated at room temperature for 30 min. The serum was removed, and the wells were washed 2 times with PBS. Horseradish peroxidase-conjugated anti-IgG antibody was added to the wells, the plate was incubated for 30 min at room temperature, and the wells were washed 2 times with PBS. 3,3′,5,5′-Tetramethylbenzidine was added to the wells, and the plate was incubated for 10 min and then read with a spectrophotometer plate reader. The results were evaluated based on the sample mean/negative-control mean (*S*/*N*) ratio.

### Statistical analyses.

Data sets were analyzed using a two-way analysis of variance (ANOVA) (no repeated measures [RM]). Unless otherwise stated in the figure legends, data are presented as mean values with standard deviations. Kaplan-Meier survival curves were analyzed for significance using the log rank test. No data sets that were analyzed for statistical significance varied in sample size by more than 3-fold.

### Ethics statement.

The animal studies were approved by the University of Florida Institutional Animal Care and Use Committee (IACUC) and were performed in accordance with the approved protocol (protocol 0541). All experiments were carried out in adherence to the Guide for the Care and Use of Laboratory Animals of the National Institutes of Health and the American Veterinary Medical Association Guidelines on Euthanasia.
